# Gastric cancer with p53 overexpression has high potential for metastasising to lymph nodes.

**DOI:** 10.1038/bjc.1993.108

**Published:** 1993-03

**Authors:** Y. Kakeji, D. Korenaga, S. Tsujitani, H. Baba, H. Anai, Y. Maehara, K. Sugimachi

**Affiliations:** Department of Surgery II, Faculty of Medicine, Kyushu University, Fukuoka, Japan.

## Abstract

**Images:**


Br. J. Cancer (1993), 67, 589-593 © Macmillan Press Ltd., 1993

### Gastric cancer with p53 overexpression has high potential for metastasising to lymph nodes

Y. Kakeji, D. Korenaga, S. Tsujitani, H. Baba, H. Anai, Y. Maehara & K. Sugimachi

*Department of Surgery II, Faculty of Medicine, Kyushu University, Fukuoka, Japan.*

**Summary** Overexpression of the tumour suppressor gene p53 was investigated immunohistochemically in 96 primary gastric carcinomas and 26 corresponding metastatic perigastric lymph nodes. Abnormalities in p53 expression were found in 52 (54%) of the 96 primary carcinomas. Tumours stained positively for p53 frequently metastasised to lymph nodes (the metastatic rate: 85%) compared to findings in those with negative p53 staining (64%, $P<0.05$). Ninety-two percent (24/26) of the malignant cells in the lymph nodes stained positively for p53. When the DNA ploidy pattern of the tumour was determined by flow cytometry, the aneuploid tumours in p53 positive and negative groups accounted for 69% and 45%, respectively ($P<0.05$). Proliferative activity of the tumour, as measured by Ki-67 labelling, was significantly higher (30.6 ± 12.0%) in the p53 positive group than that (25.1 ± 10.7%) in the p53 negative group ($P<0.05$). Thus, gastric cancer with a mutant p53 has high proliferative activity and metastasis to lymph nodes will probably occur.

Cancer occurs when there are multiple mutations in genes (Cairns, 1981; Bishop, 1987). Some classes of genes normally function to prevent or suppress tumourigenesis and inactivation of such genes leads to an oncogenic state (Hollingsworth & Lee, 1991). The p53 gene is one of these 'tumour suppressor genes', and p53 is a 53 kD nuclear protein which seems to regulate entry into the progression through the normal cell cycle (Mercer *et al.*, 1984). Studies of p53 expression in cultured cells suggested that increased levels are associated either with an abnormal mutated protein (Finlay *et al.*, 1988), or with stabilisation of the protein in a complex with viral antigens, e.g. SV40 large T antigen (Lane & Crawford, 1979). Mutation of the p53 gene at a highly conserved sequence and alteration in the expression of p53 protein are frequent occurrences in human malignancies (Nigro *et al.*, 1989; Bartek *et al.*, 1990; Rodrigues *et al.*, 1990; Iggo *et al.*, 1990).

There are basic data on p53 gene alterations in specimens of gastric cancer (Tamura *et al.*, 1991; Kim *et al.*, 1991; Yamada *et al.*, 1991). We examined the content of mutant p53 protein immunohistochemically using a large number of clinical samples, and associations with clinicopathological features were analysed. To clarify characteristics of the p53 positive tumour cells, we also analysed DNA ploidy patterns and cell proliferative activity determined by Ki-67 (Gerdes *et al.*, 1983) staining, a monoclonal antibody that recognises a human nuclear antigen expressed by proliferating cells.

### Materials and methods

### *Patients*

This study included 96 unselected patients with primary gastric cancer, all of whom underwent gastrectomy with lymph node dissection in the Department of Surgery II in Kyushu University Hospital and affiliated hospitals in Fukuoka, Japan, from 1989 to 1990. No patient had been treated preoperatively with cytotoxic drugs. Histology of excised tissues were examined in hematoxylin and eosin (H&E) stained preparations and classification was made according to the criteria of the Japanese Research Society for Gastric Cancer (1981).

Correspondence: Y. Kakeji, Department of Surgery II, Faculty of Medicine, Kyushu University, Fukuoka 812, Japan.
Received 27 May 1992; and in revised form 3 September 1992.

### *Tissue samples*

We sampled both the deep periphery of the tumour and adjacent uninvolved tissue, and from each case one representative large perigastric lymph node was sampled. Of all 96 cases, 26 metastatic lymph nodes could be investigated for p53 staining. Tissue samples for p53 and Ki-67 staining from 96 primary tumours and 26 metastatic lymph nodes were fixed in periodate-lysine-paraformaldehyde (PLP) for 5 h immediately after surgical resection, embedded in OCT compound (Miles, Elkhart) and preserved at −80°C. Six μm sections were cut on cryostat. For DNA ploidy analysis, samples taken from the same specimen used for histological staining were used fresh or were frozen without fixation at −80°C and used within 48 h.

### *Immunohistochemical staining of p53 and Ki-67*

PAb240 (Gannon *et al.*, 1990) is a mouse monoclonal antibody that recognises an evolutionarily conserved epitope on p53, the epitope lies between amino acids 156 and 214 on murine p53. This antibody reacted with human, mouse, rat, hamster, bovine and chicken p53 in Western immunoblotting experiments (Gannon *et al.*, 1990). The avidin-biotin-peroxidase complex (ABC) method was used for staining. Sections were first washed in phosphate-buffered saline, pH 7.2 (PBS), and then incubated at room temperature with normal horse serum (Vector Laboratories, Burlingame, CA: 1:10 for 15 min). We then added PAb240 (Oncogene Science, New York: 1:50 overnight), biotinylated horse anti-mouse IgG (Vector Laboratories, Burlingame CA: 1:200 for 30 min), Avidin-biotin-peroxidase complex (Vector Laboratories: for 30 min). Peroxidase labelling was developed with a diaminobenzidine (DAB) and hydrogen peroxidase ($H_2O_2$), and the sections were counterstained with Mayer's hematoxylin. We used KUPL40 (Shirasawa *et al.*, 1991) as the positive control, a carcinoma from a patient with familial polyposis coli transplanted into nude mice and known to contain a mutation in the p53 gene, detected by sequencing. Omission of the primary antibody served as the negative control.

To measure the proliferative activity, we used mouse monoclonal Ki-67 antibody (Dako, Copenhagen, Denmark: 1:100 overnight), then the procedures described above and the ABC method. We examined 1,000 nuclei in areas of the section with the highest labelling rates.

### *Flow cytometric analysis of the DNA ploidy pattern*

Preparation of cell suspensions and measurement of nuclear fluorescence were done as described by Sasaki *et al.* (1991). Cellular DNA content was measured using a FACscan flow cytometer (Becton-Dickinson Immunocytometry Systems, Mountain View, CA). The fluorescent signals from 10,000 cells were collected and the results displayed in the form of a frequency distribution histogram (DNA histogram). A diploid pattern of the tumour cells was defined as a single $G_0/G_1$ peak on the DNA histogram and an aneuploid pattern as at least one other clearly distinct $G_0/G_1$ peak followed by a small $G_2+M$ peak. The DNA index (DI) was defined as the ratio of the peak channel number of the $G_0/G_1$ tumour population divided by the peak channel number of $G_0/G_1$ diploid reference population. Data with a coefficient of variation (C.V.) over 8% and/or with excessive debris were excluded from study. The mean C.V. was 4.28 ± 1.82%.

*Statistical analysis*

Clinicopathological data was stored in an IBM 4381 mainframe computer. The Biomedical Computer Program (BMDP) was used for all statistical analyses (Dixon, 1988). The information used included the sex, age, tumour location and size, gross appearance according to the Borrman classification, degree of gastric wall invasion, status of lymph node metastatsis, and pathological class. The BMDP P4F and P3S programs were used for the chi-square test and the Mann-Whitney test to compare characteristics between p53 positive and negative groups. Quantitative data on the DNA index and Ki-67 proliferative activity were compared using Student's *t* test.

### Results

*p53 and clinicopathological features*

We stained both the deep periphery of the tumour and adjacent tumour-free tissue. A distinct nuclear immunoreaction for p53 was judged as positive. In the positive cells, the

**Figure 1** **a**, Photomicrograph of primary gastric cancer as seen in a frozen section stained using monoclonal antibody PAb240. Note the homogenous, positive nuclear staining ( × 100). **b**, Metastatic cancer in a perigastric lymph node. There is a wide-spread overexpression of p53 yet there was no detectable expression of p53 in the primary tumour ( × 100).

nuclear staining pattern was diffuse with little variation (Figure 1a). Cytoplasmic staining was seen in a few cases but the intensity was weak. Heterogeneity of p53 staining in one specimen of each case was recognised in 9 (9.4%) cases. Though almost all positive cells formed clusters, for only one case was there sporadic staining (1%) of cancer cells and here there was no vessel invasion or lymph node metastasis. Samples in wich rare nuclei exhibited positive staining for p53 or samples with weak reactivity were considered to express normal levels of p53 (Davidoff *et al.*, 1991*a*,*b*; Isola *et al.*, 1992; Hiyoshi *et al.*, 1992). Case with over 10% cancer cells showing positive nuclear staining were defined as 'positive-staining'. Positive p53 staining was evident in 52 (54%) of 96 primary gastric tumours. The surrounding gastric tissue with no histological evidence of malignant invasion did not stain.

Table I shows clinicopathologic characteristics and p53 overexpression. There was no obvious relation between p53 staining and the sex or age of the patient. Association with histologic type was not statistically significant, though cancer cells with a tubular or medullary formation often stained positive for p53. Some early stage gastric cancers stained positively for p53, the staining being similar in proportion to tumours showing invasion into deeper layers. However, a significant association was found between p53 overexpression and the metastatic spread to lymph nodes. p53-positive tumours were associated with a higher incidence of metastasis to lymph nodes (85%) than were p53-negative (64%, $P<0.05$). A p53-positive gastric cancer correlated with a higher rate of venous invasion (88%), compared with the p53-negative isolates (68%, $P<0.05$). As for metastasis to the liver, and peritoneal dissemination, there were no statistical differences.

We also investigated 26 metastatic lymph nodes using PAb240 (Table II). The majority of the cancer cells in the metastatic lymph nodes stained positively for p53. In 17 of 18 cases with a p53-positive primary and in 7 of 8 with a p53 negative primary tumour, the metastatic nodes stained positively for p53 (Figure 1b).

As for the prognosis, the follow-up period was only 1–3 years, however, at this writing we found no significant differences between p53-positive and negative groups (data not shown).

### *p53 and DNA ploidy or proliferative activity*

p53-positive isolates showed a significantly higher incidence of aneuploid pattern (69%) than did p53-negative isolates (45%, $P<0.05$, Table III). The mean DNA index for the

**Table I** p53 overexpression and clinicopathological characteristics

| | *p53 staining* Negative n(%) | Positive n(%) | P value |
|---|---|---|---|
| 1 Sex | | | |
| Men | 31 (70) | 33 (63) | N.S.[a] |
| Women | 13 (30) | 19 (37) | |
| 2 Age, y (mean ± s.d.) | 64.1 ± 10.8 | 62.4 ± 12.0 | N.S. |
| 3 Location of tumour | | | |
| Upper (C) | 8 (18) | 6 (12) | N.S. |
| Middle (M) | 5 (11) | 8 (15) | |
| Lower (A) | 31 (70) | 37 (71) | |
| 4 Tumour size, cm (mean ± s.d.) | 8.1 ± 3.9 | 8.1 ± 4.1 | N.S. |
| 5 Gross appearance | | | |
| Superficial | 6 (14) | 2 (4) | N.S. |
| Localised | 18 (41) | 16 (31) | |
| Infiltrative | 18 (41) | 30 (58) | |
| Unclassified | 2 (4) | 4 (7) | |
| 6 Histologic type | | | |
| Papillary | 0 (0) | 2 (4) | N.S. |
| Well | 4 (9) | 9 (17) | |
| Moderately | 14 (32) | 15 (29) | |
| Poorly | 20 (45) | 24 (46) | |
| Signet | 3 (7) | 0 (0) | |
| Mucinous | 3 (7) | 2 (4) | |
| 7 Mode of invasion | | | |
| Expansive | 9 (20) | 6 (12) | N.S. |
| Intermediate | 17 (39) | 23 (44) | |
| Infiltrative | 13 (30) | 18 (35) | |
| 8 Depth of cancer invasion[b] | | | |
| m, sm | 6 (14) | 5 (10) | N.S. |
| pm, ss | 11 (25) | 14 (28) | |
| se, si | 27 (61) | 33 (63) | |
| 9 Invasion into lymphatics | | | |
| Negative | 5 (11) | 3 (6) | N.S. |
| Positive | 39 (89) | 49 (94) | |
| 10 Metastases to the lymph nodes | | | |
| Negative | 16 (36) | 8 (15) | $P<0.05$ |
| Positive | 28 (84) | 44 (85) | |
| 11 Venous invasion | | | |
| Negative | 14 (32) | 6 (12) | $P<0.05$ |
| Positive | 30 (68) | 46 (88) | |
| 12 Metastasis to the liver | | | |
| Negative | 40 (91) | 46 (88) | N.S. |
| Positive | 4 (9) | 6 (12) | |
| 13 Peritoneal dissemination | | | |
| Negative | 40 (91) | 46 (88) | N.S. |
| Positive | 4 (9) | 6 (12) | |
| Total | 44 (100) | 52 (100) | |

[a]Not significant; [b]Depth of invasion. m: musoca; sm: submucosa; pm: muscularis propria; ss: cancer cells extend to subserosa; se: cancer cells present on the serosal surface and exposed to the peritoneal cavity; si: cancer cells infiltrating neighbouring tissue.

**Table II** p53 overexpression in primary tumour and metastatic lymph nodes

| *Primary tumour* | | | *Metastatic lymph node* |
|---|---|---|---|
| p53 (−) | 1 | 1 | p53 (−) |
| | 7 | 1 | |
| p53 (+) | 1 | 7 | p53 (+) |
| | 17 | 17 | |

**Table III** p53 overexpression, DNA ploidy, and proliferative activity

| | *p53 staining* | | |
|---|---|---|---|
| | *Negative* | *Positive* | *P value* |
| DNA ploidy | | | |
| Diploid | 24 (55%) | 16 (31%) | $P<0.05$ |
| Aneuploid | 20 (45%) | 36 (69%) | |
| DNA index (mean ± s.d.) | 1.19 ± 0.26 | 1.30 ± 0.28 | $P<0.05$ |
| Ki-67 labelling percentage (mean ± s.d.) | 25.1 ± 10.7% | 30.6 ± 12.0% | $P<0.05$ |

p53-positive cases is 1.30, a value significantly higher than that (1.19) for the p53-negative cases ($P<0.05$).

The proliferative activity expressed by mean Ki-67 labelling percent was 30.6% for p53-positive cases, a value significantly higher than 25.1% for the p53-negative cases ($P<0.05$).

### Discussion

In the present immunohistochemical study of human gastric cancer, we found that some diploid tumours or early stage gastric cancers showed an overexpression of p53, hence the possibility that mutation in this gene may occur, even in the early stage of cancer progression has to be considered. This notion is also supported by the finding that p53 mutations were involved in the formation of both carcinomas and adenomas which occur in familial polyposis coli (Shirasawa *et al.*, 1991).

Histologically, abnormal p53 protein expression was often recognised in cancer cells in tubular or medullar formation regardless of predominant histologic type. These findings are in close agreement with the report of Yasui *et al.* (1991), in which loss of heterozygosity on chromosome 5q and 17p was frequent in cases of well differentiated types of gastric cancer.

The relationship between p53 overexpression and metastatic spread to lymph nodes was noted in breast cancer (Davidoff *et al.*, 1991*a*). In our study, there was a significant association between overexpression of p53 in gastric cancer and lymph node metastasis. Tumours with positive p53 staining frequently metastasised to lymph nodes and most metastatic lesions showed positive p53 staining. Furthermore, tumours with positive p53 staining had a higher proliferative activity than did those with which stained negatively. Cattoretti *et al.* (1988) reported a correlation between p53 immunoreaction and the Ki-67 score in cases of breast cancer. We also reported data that tumours with a high proliferative activity often metastasised to lymph nodes (Kakeji *et al.*, 1991). There are findings that nuclear phosphoprotein p53 is expressed in all cells late in the $G_1$ phase of the cycle and may regulate entry of the cells into the S phase (DNA synthesis) (Shohat *et al.*, 1987). Hence failure to appropriately regulate p53 expression may lead to an uncontrolled cell growth. In light of all these observations, the mutation of p53 seems to lead to activation of cell proliferation and the risk or lymph node metastasis is increased. The over-expressed mutant p53 is not merely a remnant of the mutational inactivation of p53 suppressor function, but also actively promotes growth of the tumour. As shown in Table II, positive staining for p53 occurred only in the lymph nodes, in seven cases. As staining for p53 was positive in the early stage gastric cancers and there was some heterogeneity in this staining, the primary lesion may have contained a subpopulation of the mutant p53.

As for prognosis and p53 stainability, Scott *et al.* (1991) reported no difference in cases of colorectal carcinoma, however, Iwaya *et al.* (1991) did find differences in cases of breast carcinoma. Though we found no significant difference between p53 positive and negative tumours with regard to 2-year or 3-year survival rates, a longer follow-up is needed before conclusions can be reached.

Considering the association between genetic change and protein production, most of the cancers showing nuclear p53 immunoreaction also carried a mutation of the p53 gene, as noted in studies of breast, colorectal and lung cancer cell lines and tissues (Bartek *et al.*, 1990; Rodrigues *et al.*, 1990; Iggo *et al.*, 1990; Davidoff *et al.*, 1991*b*). Though the number was small, Seruca *et al.* (1992) reported the association and some discrepancy between immunoreactivity and mutations in gastric cancer. Immunohistochemical analysis of malignant tissues appears to be a valid method which can be used to screen for the presence of mutant p53, however, it does have limitations. Data on the immunohistochemistry of p53 in gastric cancer may be clinically useful for getting some information on the metastatic potential to lymph nodes.

We thank Drs M. Furusawa, M. Saku, and S. Shirasawa and associated surgeons for providing tissue specimens, and M. Ohara for helpful comments.

### References

BARTEK, J., IGGO, R., GANNON, J. & LANE, D.P. (1990). Genetic and immunochemical analysis of mutant p53 in breast cancer cell line. *Oncogene*, **5**, 893–899.

BISHOP, J.M. (1987). The molecular genetics of cancer. *Science*, **235**, 305–311.

CAIRNS, J. (1981). The origin of human cancers. *Nature*, **289**, 353–357.

CATTORETTI, G., RILKE, R., ANDREOLA, S., D'AMATO, L. & DELIA, D. (1988). p53 expression in breast cancer. *Int. J. Cancer*, **41**, 178–183.

DAVIDOFF, A.M., HERNDON II, J.E., GLOVER, N.S., KERNS, B.J.M., PENCE, J.C., IGLEHART, J.D. & MARKS, J.R. (1991*a*). Relation between p53 overexpression and established prognostic factors in breast cancer. *Surgery*, **110**, 259–264.

DAVIDOFF, A.M., HUMPHERY, P.A., IGLEHART, J.D. & MARKS, J.R. (1991*b*). Genetic basis for p53 overexpression in human breast cancer. *Proc. Natl Acad. Sci. USA*, **88**, 5006–5010.

DIXON, W.J. (1988) ed. *BMDP Statistical Software Manual*. pp. 13–744. Berkeley: University of California Press.

FINLAY, C.A., HINDS, P.W., TAN, T.H., ELIYAHU, D., OREN, M. & LEVINE, A.J. (1988). Activating mutations for transformation by p53 produce a gene product that forms an hsc 70-p53 complex with an altered half life. *Mol. Cell Biol.*, **8**, 531–539.

GANNON, J.V., GREAVES, R., IGGO, R. & LANE, D.P. (1990). Activating mutations in p53 produce a common conformational effect. A monoclonal antibody specific for the mutant form. *EMBO J.*, **9**, 1595–1602.

GERDES, J., SCHWAB, U., LEMKE, H. & STEIN, H. (1983). Production of a mouse monoclonal antibody reactive with a human nuclear antigen associated with cell proliferation. *Int. J. Cancer*, **31**, 13–20.

HIYOSHI, I., MATSUNO, Y., KATO, H., SHIMOSATO, Y. & HIROHASHI, S. (1992). Clinicopathological significance of nuclear accumulation of tumor suppressor gene p53 product in primary lung cancer. *Jpn. J. Cancer Res.*, **83**, 101–106.

HOLLINGSWORTH, R.E. & LEE, W.H. (1991). Tumor suppressor genes: new prospects for cancer research. *J. Natl Cancer Inst.*, **83**, 91–96.

IGGO, R., GATTER, K., BARTEK, J., LANE, D. & HARRIS, A.L. (1990). Increased expression of mutant forms of p53 oncogene in primary lung cancer. *Lancet*, **35**, 675–679.

ISOLA, J., VISACORPI, T., HOLLI, K. & KALLIONIEMI, O.P. (1992). Association of overexpression of tumor suppressor protein p53 with rapid cell proliferation and poor prognosis in node-negative breast cancer patients. *J. Natl Cancer Inst.*, **84**, 1109–1114.

IWAYA, K., TSUDA, H., HIRAIDE, H., TAMAKI, K., TAMAKUMA, S., FUKUTOMI, T., MUKAI, K. & HIROHASHI, S. (1991). Nuclear p53 immunoreaction associated with poor prognosis of breast cancer. *Jpn. J. Cancer Res.*, **82**, 835–840.

JAPANESE RESEARCH SOCIETY FOR GASTRIC CANCER (1981). The general rules for the gastric cancer study in surgery and pathology. *Jpn. J. Surg.*, **11**, 127–145.

KAKEJI, Y., KORENAGA, D., TSUJITANI, S., HARAGUCHI, M. MAEHARA, Y. & SUGIMACHI, K. (1991). Predictive value of Ki-67 and argyrophilic nucleolar organizer region staining for lymph node metastasis in gastric cancer. *Cancer Res.*, **51**, 3503–3506.

KIM, J.H., TAKAHASHI, T., CHIBA, I., PARK, J.G., BIRRER, M.J., ROH, J.K., LEE, H.D., KIM, J.P., MINNA, J.D. & GAZDAR, A.F. (1991). Occurrence of p53 gene abnormalities in gastric carcinoma tumors and cell lines. *J. Natl Cancer Inst.*, **83**, 938–943.

LANE, D.P. & CRAWFORD, L.V. (1979). T antigen is bound to a host protein in SV40-transformed cells. *Nature*, **278**, 261–263.

MERCER, W.E., AVIGNOLO, C. & BASERGA, R. (1984). Role of the p53 protein in cell proliferation as studied by microinjection of monoclonal antibodies. *Mol. Cell Biol.*, **4**, 276–281.

NIGRO, J.M., BAKER, S.J., PREISINGER, A.C., JESSUP, J.M., HOSTETTER, R., CLEARY, K., BIGNER, S.H., DAVIDSON, N., BAYLIN, S., DEVILEE, P., GLOVER, T., COLLINS, F.S., WESTON, A. MODALI, R., HARRIS, C.C. & VOGELSTEIN, B. (1989). Mutations in the p53 gene occur in diverse human types. *Nature*, **342**, 705–708.

RODRIGUES, N.R., ROWAN, A., SMITH, M.E.F., KERR, I.B., BODMER, W.F., GANNON, J.V. & LANE, D.P. (1990). p53 mutation in colorectal cancer. *Proc. Natl Acad. Sci. USA*, **87**, 7555–7559.

SASAKI, K., MURAKAMI, T., MURAKAMI, T. & NAKAMURA, M. (1991). Intratumoral heterogeneity in DNA ploidy of eosphageal squamous cell carcinomas. *Cancer*, **68**, 2403–2406.

SCOTT, N., SAGAR, P., STEWART, J., BLAIR, G.E., DIXON M.F. & QUIRKE, P. (1991). p53 in colorectal cancer: clinicopathological correlation and prognostic significance. *Br. J. Cancer*, **63**, 317–319.

SERUCA, R., DAVID, L., HOLM, R., NESLAND, J.M., FANGAN, B.M., CASTEDO, S., SIMOES, M.S. & BORRESEN, A.L. (1992). p53 mutations in gastric carcinomas. *Br. J. Cancer*, **65**, 708–710.

SHIRASAWA, S., URABE, K., YANAGAWA, Y., TOSHITANI, K., IWAMA, T. & SASAZUKI, T. (1991). p53 gene mutations in colorectal tumors from patients with familial polyposis coli. *Cancer Res.*, **51**, 2874–2878.

SHOHAT, O., GREENBERG, M., REISMAN, D., OREN, M. & ROTER, V. (1987). Inhibition of cell growth mediated by plasmids encoding p53 antisense. *Oncogene*, **1**, 277–283.

TAMURA, G., KIHARA, T., NOMURA, K., TERADA, M., SUGIMURA, T. & HIROHASHI, S. (1991). Detection of frequent p53 gene mutations in primary gastric cancer by cell sorting and polymerase chain reaction single-strand conformation polymorphism analysis. *Cancer Res.*, **51**, 3056–3058.

YAMADA, Y., YOSHIDA, T., HAYASHI, K., SEKIYA, T., YOKOTA, J., HIROHASHI, S., NAKATANI, K., NAKANO, H., SUGIMURA, T. & TERADA, M. (1991). p53 gene mutations in gastric cancer metastases and in gastric cancer cell lines derived from metastases. *Cancer Res.*, **51**, 5800–5805.

YASUI, W., YOSHIDA, K., ITO, H. & TAHARA, E. (1991). Molecular diagnosis in gastric cancer. *Jpn. J. Cancer Chemother.*, **18**, 7–13 (in Japanese with English summary).


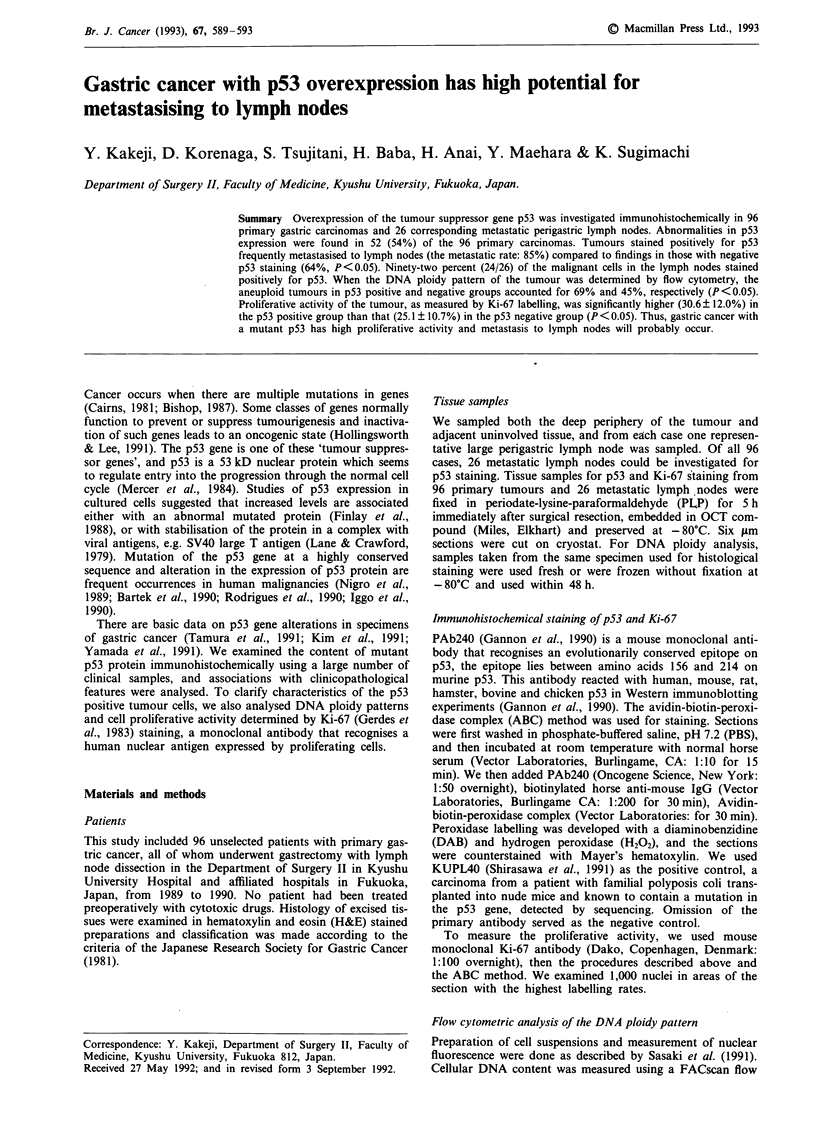

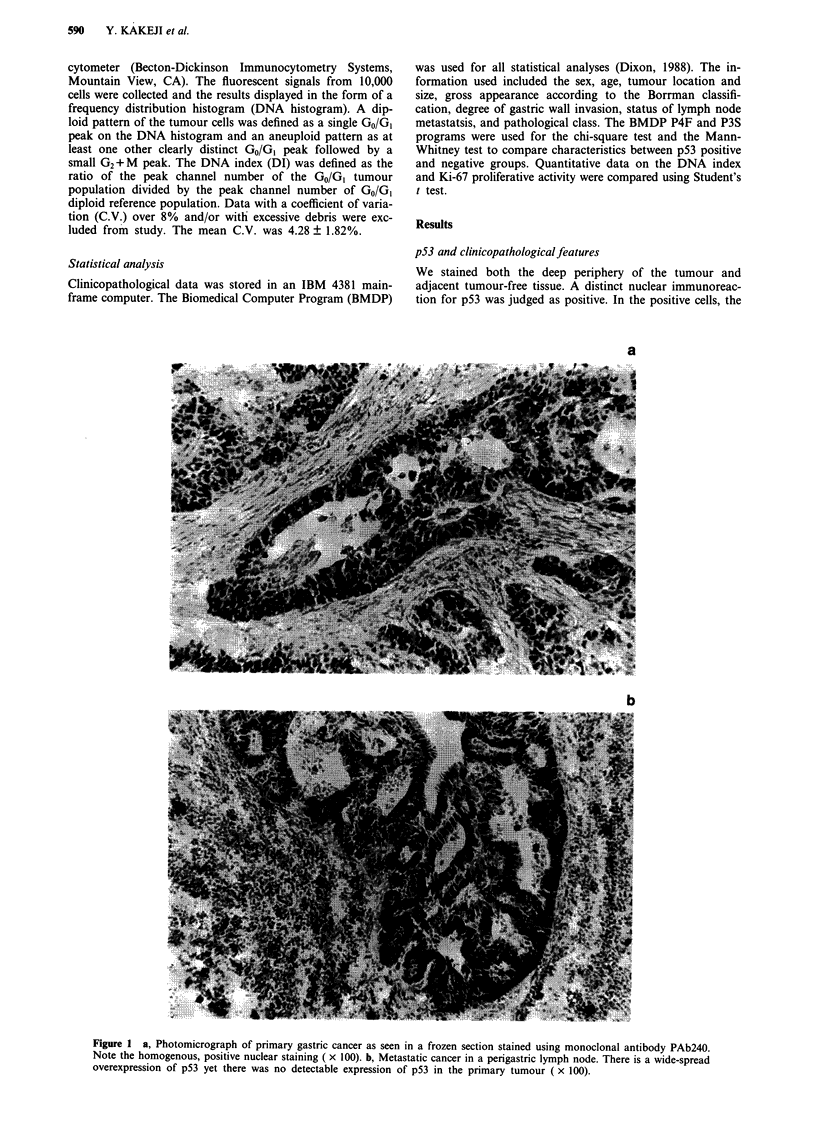

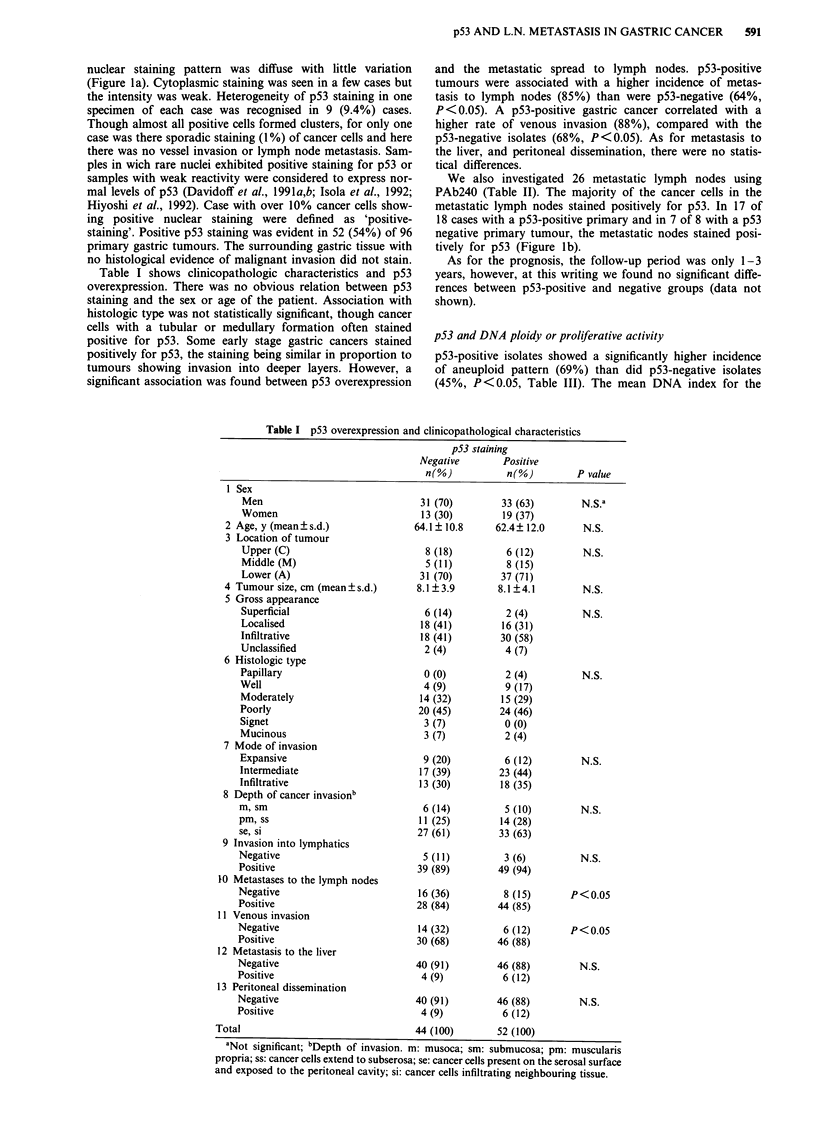

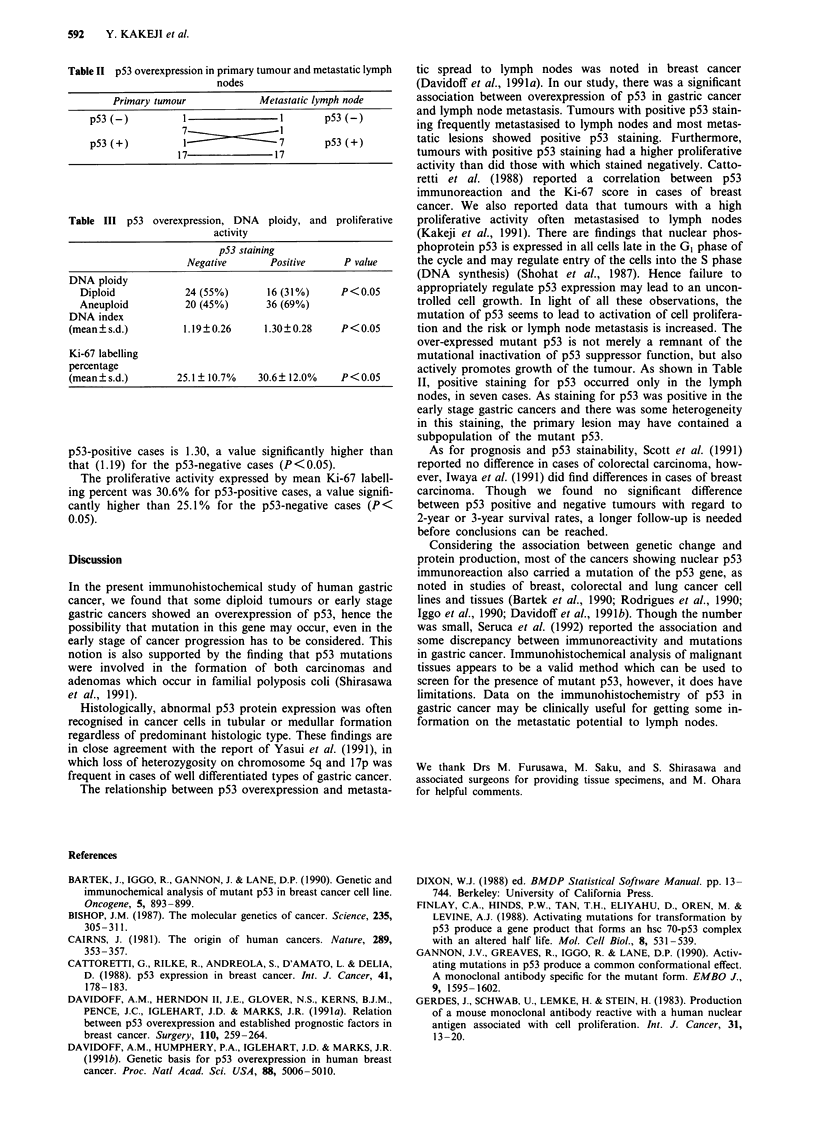

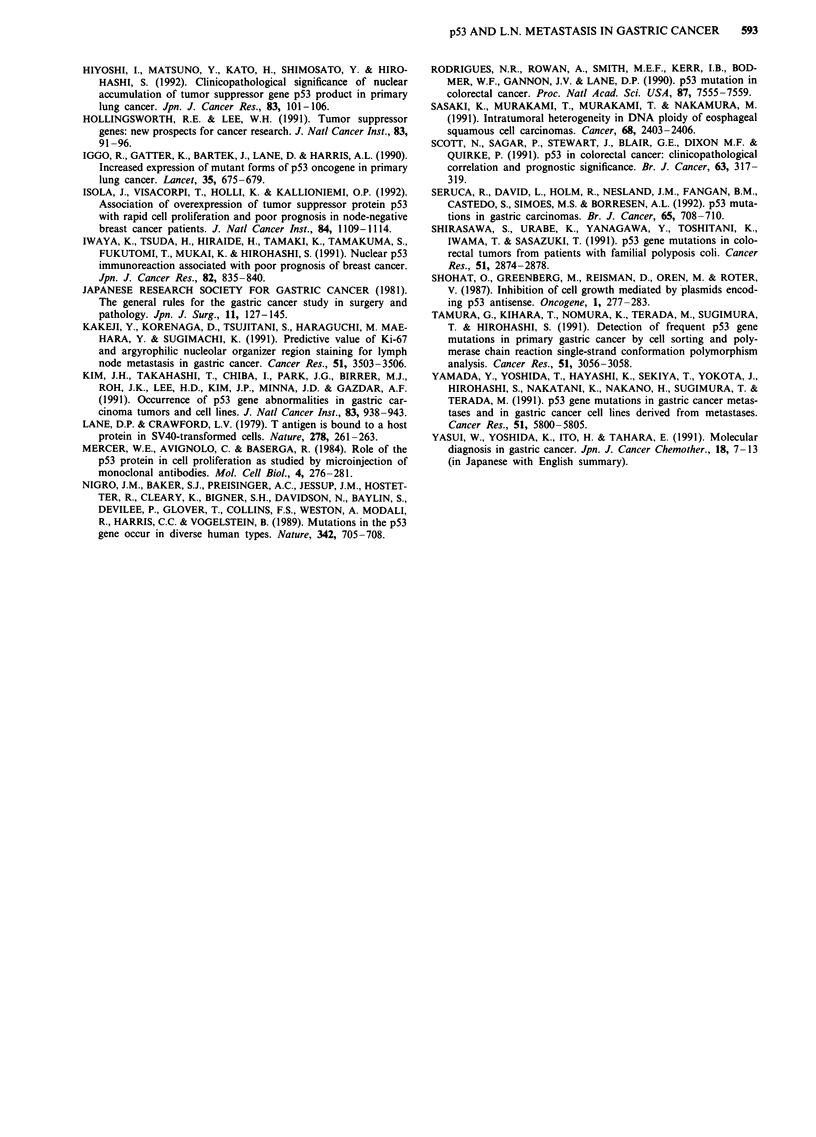


## References

[OCR_00425] Bartek J., Iggo R., Gannon J., Lane D. P. (1990). Genetic and immunochemical analysis of mutant p53 in human breast cancer cell lines.. Oncogene.

[OCR_00430] Bishop J. M. (1987). The molecular genetics of cancer.. Science.

[OCR_00434] Cairns J. (1981). The origin of human cancers.. Nature.

[OCR_00438] Cattoretti G., Rilke F., Andreola S., D'Amato L., Delia D. (1988). P53 expression in breast cancer.. Int J Cancer.

[OCR_00445] Davidoff A. M., Herndon J. E., Glover N. S., Kerns B. J., Pence J. C., Iglehart J. D., Marks J. R. (1991). Relation between p53 overexpression and established prognostic factors in breast cancer.. Surgery.

[OCR_00449] Davidoff A. M., Humphrey P. A., Iglehart J. D., Marks J. R. (1991). Genetic basis for p53 overexpression in human breast cancer.. Proc Natl Acad Sci U S A.

[OCR_00458] Finlay C. A., Hinds P. W., Tan T. H., Eliyahu D., Oren M., Levine A. J. (1988). Activating mutations for transformation by p53 produce a gene product that forms an hsc70-p53 complex with an altered half-life.. Mol Cell Biol.

[OCR_00464] Gannon J. V., Greaves R., Iggo R., Lane D. P. (1990). Activating mutations in p53 produce a common conformational effect. A monoclonal antibody specific for the mutant form.. EMBO J.

[OCR_00470] Gerdes J., Schwab U., Lemke H., Stein H. (1983). Production of a mouse monoclonal antibody reactive with a human nuclear antigen associated with cell proliferation.. Int J Cancer.

[OCR_00480] Hiyoshi H., Matsuno Y., Kato H., Shimosato Y., Hirohashi S. (1992). Clinicopathological significance of nuclear accumulation of tumor suppressor gene p53 product in primary lung cancer.. Jpn J Cancer Res.

[OCR_00484] Hollingsworth R. E., Lee W. H. (1991). Tumor suppressor genes: new prospects for cancer research.. J Natl Cancer Inst.

[OCR_00489] Iggo R., Gatter K., Bartek J., Lane D., Harris A. L. (1990). Increased expression of mutant forms of p53 oncogene in primary lung cancer.. Lancet.

[OCR_00494] Isola J., Visakorpi T., Holli K., Kallioniemi O. P. (1992). Association of overexpression of tumor suppressor protein p53 with rapid cell proliferation and poor prognosis in node-negative breast cancer patients.. J Natl Cancer Inst.

[OCR_00500] Iwaya K., Tsuda H., Hiraide H., Tamaki K., Tamakuma S., Fukutomi T., Mukai K., Hirohashi S. (1991). Nuclear p53 immunoreaction associated with poor prognosis of breast cancer.. Jpn J Cancer Res.

[OCR_00513] Kakeji Y., Korenaga D., Tsujitani S., Haraguchi M., Maehara Y., Sugimachi K. (1991). Predictive value of Ki-67 and argyrophilic nucleolar organizer region staining for lymph node metastasis in gastric cancer.. Cancer Res.

[OCR_00516] Kim J. H., Takahashi T., Chiba I., Park J. G., Birrer M. J., Roh J. K., De Lee H., Kim J. P., Minna J. D., Gazdar A. F. (1991). Occurrence of p53 gene abnormalities in gastric carcinoma tumors and cell lines.. J Natl Cancer Inst.

[OCR_00521] Lane D. P., Crawford L. V. (1979). T antigen is bound to a host protein in SV40-transformed cells.. Nature.

[OCR_00525] Mercer W. E., Avignolo C., Baserga R. (1984). Role of the p53 protein in cell proliferation as studied by microinjection of monoclonal antibodies.. Mol Cell Biol.

[OCR_00530] Nigro J. M., Baker S. J., Preisinger A. C., Jessup J. M., Hostetter R., Cleary K., Bigner S. H., Davidson N., Baylin S., Devilee P. (1989). Mutations in the p53 gene occur in diverse human tumour types.. Nature.

[OCR_00539] Rodrigues N. R., Rowan A., Smith M. E., Kerr I. B., Bodmer W. F., Gannon J. V., Lane D. P. (1990). p53 mutations in colorectal cancer.. Proc Natl Acad Sci U S A.

[OCR_00542] Sasaki K., Murakami T., Murakami T., Nakamura M. (1991). Intratumoral heterogeneity in DNA ploidy of esophageal squamous cell carcinomas.. Cancer.

[OCR_00549] Scott N., Sagar P., Stewart J., Blair G. E., Dixon M. F., Quirke P. (1991). p53 in colorectal cancer: clinicopathological correlation and prognostic significance.. Br J Cancer.

[OCR_00553] Seruca R., David L., Holm R., Nesland J. M., Fangan B. M., Castedo S., Sobrinho-Simões M., Børresen A. L. (1992). P53 mutations in gastric carcinomas.. Br J Cancer.

[OCR_00558] Shirasawa S., Urabe K., Yanagawa Y., Toshitani K., Iwama T., Sasazuki T. (1991). p53 gene mutations in colorectal tumors from patients with familial polyposis coli.. Cancer Res.

[OCR_00564] Shohat O., Greenberg M., Reisman D., Oren M., Rotter V. (1987). Inhibition of cell growth mediated by plasmids encoding p53 anti-sense.. Oncogene.

[OCR_00569] Tamura G., Kihana T., Nomura K., Terada M., Sugimura T., Hirohashi S. (1991). Detection of frequent p53 gene mutations in primary gastric cancer by cell sorting and polymerase chain reaction single-strand conformation polymorphism analysis.. Cancer Res.

[OCR_00576] Yamada Y., Yoshida T., Hayashi K., Sekiya T., Yokota J., Hirohashi S., Nakatani K., Nakano H., Sugimura T., Terada M. (1991). p53 gene mutations in gastric cancer metastases and in gastric cancer cell lines derived from metastases.. Cancer Res.

[OCR_00583] Yasui W., Yoshida K., Ito H., Tahara E. (1991). [Molecular diagnosis of gastric cancer].. Gan To Kagaku Ryoho.

